# Availability of Nitrogen in Soil for Irrigated Cotton Following Application of Urea and 3,4-Dimethylpyrazole Phosphate-Coated Urea in Concentrated Bands

**DOI:** 10.3390/plants12051170

**Published:** 2023-03-03

**Authors:** Pamela A. Pittaway, Diogenes L. Antille, Alice R. Melland, Serhiy Marchuk

**Affiliations:** 1 Centre for Agricultural Engineering, University of Southern Queensland, Toowoomba, QLD 4350, Australia; 2 CSIRO Agriculture and Food, Black Mountain Science and Innovation Precinct, Canberra, ACT 2601, Australia

**Keywords:** dissolved organic nitrogen, enhanced efficiency fertilizers, exchangeable ammonium, irrigated Vertisol, nitrogen fertilizer use efficiency, nitrification inhibition, root exclusion tubes

## Abstract

Low nitrogen (N) fertilizer use efficiency for irrigated cotton has been attributed to the limited ability of tap roots to access N from concentrated subsurface bands, or the preferential root uptake of microbially-mineralized dissolved organic N. This work investigated how applying high-rate banded urea affects the availability of N in soil and the capacity of cotton roots to take up N. Soil was analyzed for water-extractable total dissolved N and inorganic N species after urea or urea coated with 3,4-dimethylpyrazole phosphate (DMPP) was applied at concentrations of 261, 455, 461, and 597 mg N kg^−1^ of (air-dry) soil (mean bulk density: 1.01 g cm^−3^). A mass balance was used to compare N applied as fertilizer and in unfertilized soil (supplied N) with the N recovered from soil within the cylinders (recovered N) at five plant growth phases. Root uptake was estimated by comparing ammonium-N (NH_4_-N) and nitrate-N (NO_3_-N) in soil sampled from within cylinders with soil sampled from immediately outside. Recovered N was up to 100% above supplied N within 30 days of applying urea above 261 mg N kg^−1^ of soil. Significantly lower NO_3_-N in soil sampled from immediately outside the cylinders suggests urea application stimulates cotton root uptake. The use of DMPP-coated urea prolonged high NH_4_-N in soil and inhibited the mineralization of released organic N. These results imply the release of previously sequestered soil organic N within 30 days of applying concentrated urea enhances the availability of NO_3_-N in the rhizosphere, reducing N fertilizer use efficiency.

## 1. Introduction

The results of isotopically labeled inorganic nitrogen (^15^N) use efficiency trials in irrigated cotton indicate that only 25% to 50% of pre-plant N fertilizer is recovered in plant biomass at harvest [1,2]. After harvest, only 10% to 30% of the ^15^N was recovered from soil, implying that 30% to 50% of the N required by the crop was supplied by soil organic N [3,4]. Some studies have postulated that cotton roots preferentially take up microbially mineralized N (e.g., [5,6]). Alternatively, the geometry of the taproot system may limit the capacity of the crop to exploit fertilizer banded into topsoil [7], with fertilizer N lost to nitrate leaching, denitrification, and nitrous oxide emissions [8,9,10,11]. Nitrification inhibitors, including 3,4-dimethylpyrazole phosphate [12] have reduced nitrous oxide and nitrate losses in broadacre agricultural systems [13,14], with no conclusive evidence of improvements in crop yield and N fertilizer use efficiency [13,15,16,17].

The relatively low N fertilizer use efficiency (<50%) reported in earlier studies (e.g., [1]) was associated with high rates of ^15^N urea applied in concentrated bands once or twice before planting or early after planting, whereas a higher efficiency (range: 58% to 93%) was achieved by applying ^15^N-labeled urea ammonium nitrate in 24 drip irrigation events over six weeks [18]. Scheduling fertilizer application with early crop N demand (canopy reflectance method) enabled the fertilizer rate to be reduced by 17 to 112 kg N ha^−1^, improving fertilizer N use efficiency without reducing crop yield. Cotton roots can extend by up to 80 mm per day, and the root system remains the dominant sink for N until early boll formation (60 to 70 days after emergence) [19]. Root growth slows with the onset of reproductive development [20], with root decomposition exceeding root extension by about 85 days after seedling emergence. Nitrate stimulates root development in cotton [21], with root volume, surface area, and root length greater than when ammonium is applied at the same N rate [19].

The objectives of this study were to investigate how a single, concentrated application of urea with and without the nitrification inhibitor 3,4-dimethylpyrazole phosphate (DMPP), affected the soil N supply available for root uptake of cotton crops during the growing season. The work reported in this article goes some way to address key knowledge gaps relating to soil N supply dynamics in Australian cropping systems [22]. Root exclusion cylinders [23] were inserted adjacent to the planting row of cotton crops to monitor the concentration of organic and inorganic N species in soil after a single, banded application of urea or DMPP-coated urea, in the presence and absence of actively growing roots. Soil sampling was timed to within three days after rainfall or irrigation when dry/wet cycles would have stimulated microbial activity [24]. Rapid analytical methods were used to minimize microbial transformations of N species during soil preparation and analysis [25]. Sampling soil from inside root exclusion cylinders should indicate the organic and inorganic N supply available to cotton plants at key crop growth stages. The difference in soil N recovered from inside and immediately outside the cylinders should indicate the capacity of cotton roots to take up N from concentrated fertilizer bands. Documenting the supply of N species in soil available for root uptake over the growing season may provide insights into why the N fertilizer use efficiency of irrigated cotton is low.

## 2. Materials and Methods

### 2.1. Experimental Site

Field trials were established on two commercial farms equipped with overhead irrigation near Pittsworth (27°42′58.33″ S, 151°37′59.68″ E, henceforth CLP) and near Yargullen (27°29′02.3″ S, 151°35′41.6″ E, henceforth NAS, INT, and SAT) in the Darling Downs region of south-east Queensland, Australia. The soils at the sites were black alkaline, self-mulching clay containing between 62% and 71% clay (<0.002 mm), 8% and 15% silt (0.002–0.02 mm), and 21% and 30% sand (0.02–2 mm) in the 0–1 m depth interval, with predominantly smectite clay minerals [26]. The soils are described in the Australian Soil Classification of Isbell [27] as Black Vertosol (Vertisol using the USDA-NRCS [28] soil description).

The 1:5 water extract pH ranged from 7.98 to 9.55 with dry soil bulk densities from 0.98 to 1.06 g cm^−3^ [29], depending on the site and measured depth interval. The effective cation exchange capacity ranged from 26.9 to 35.4 mEq 100 g^−1^, with calcium (Ca) and magnesium (Mg) as the dominant cations. A detailed description of soil properties is shown in Table 1.

In the 2016/17 season, cotton was sown at CLP and NAS on 21 October 2016 and 14 October 2016, respectively. In the 2017/18 season, cotton was sown at INT and SAT on 1 and 2 November 2017, respectively. After sufficient seedlings had emerged to establish a commercially feasible crop (≥7 plants per m), three fertilizer treatments, namely: no fertilizer (control), granular urea, and urea coated with DMPP [12] were applied. The fertilizer treatments were replicated in four 6 m plots containing three (NAS, INT, and SAT) or four (CLP) cotton rows depending upon the site (40 inch row spacing at CLP and NAS, 60 inch spacing at SAT and INT), using a planting density of 14 seeds m^−1^. Ten polyvinyl chloride cylinders (dimensions: 50 mm diameter, 300 mm long) were used as root exclusion tubes [23]. The cylinders were inserted along the fertilizer row adjacent to the plant row, within each plot. There were 40 PVC cylinders per treatment.

The fertilizer application rate in the plots was the same as the rate of N in each fertilizer band allocated per row of cotton by the farmer (two bands per plant row at CLP, and four bands per plant row at NAS, INT, and SAT). Urea or DMPP-coated urea was inserted at a depth of 50 mm within and between the root exclusion cylinders and was covered with soil to minimize volatilization (subsurface application). In the 2016/17 season, two cylinders per plot were removed within three to five days after a rainfall or irrigation event (≥20 mm of water), at five cotton growth phases (emergence, mid-squaring, peak bloom, open boll, defoliation). Soil cylinders were stored on ice during transit from the field to the laboratory, and refrigerated at 4 °C prior to sample preparation. Samples were prepared and analyzed within four days of soil collection to minimize the microbial transformation of N species during transit, storage, and preparation [25]. Soil from the two cylinders was mixed to produce a composite plot sample for chemical analysis. In the 2017/18 season, sampling was compressed to five times within the first 90 days after fertilizer application due to slightly delayed crop establishment.

### 2.2. Preparation and Chemical Analysis of Filtered Water Extracts

Five filtered water extracts were prepared from each plot sample by mixing 2 g of field-moist soil with 40 mL of deionized water on a clock-face shaker at 15 rpm for 1 h [30]. The samples were centrifuged at 2500× *g* for 5 min prior to filtration through a 0.45 μm glass fiber filter (Advantec GB-140). For the first three samplings in 2016/17, total dissolved N (TDN) was also measured on an oxidative combustion, infrared detection analyzer (Shimadzu TOC-V CSH). The alkaline potassium peroxodisulfate digestion and flow injection automated color method (Flow Injection Analyzer, Lachat Instruments) [31] was used for all subsequent samplings due to equipment failure. The ultraviolet (UV) light absorbance of water extracts measured on a Jenway 6705 UV/Vis spectrophotometer at 224 nm (UV_224_), a surrogate for NO_3_-N [32], was used to select the median sample for mineral N analysis. A modified Catchpoole and Weier [33] water pre-treatment for the 2M KCl extraction of NH_4_-N from soil was developed by adding 4M KCl 1:1 by volume to the filtered water extracts. The high osmotic potential of the solution inhibited microbial activity, reducing the likelihood of N transformations prior to analysis. In the 2016/17 sample, KCl-extractable NH_4_-N, NO_3_-N, and NO_2_-N in the water extracts were analyzed using the modified Berthelot indophenol method and NO_3_-N and NO_2_-N were analyzed using the hydrazine reaction with automated color [34]. In 2017/18, the cadmium–copper reduction method with flow injection automated color (Flow Injection Analyzer, Lachat Instruments) was used with NO_3_-N analyzed in the presence of NO_2_-N (NO*x*). The sum of 2M KCl-extractable mineral N components present in water extracts was defined as dissolved inorganic N. In 2016/17, dissolved inorganic N was calculated as the sum of NO_3_-N, NO_2_-N, and NH_4_-N, and in 2017/18 as NO*x*-N and NH_4_-N.

### 2.3. Chemical Analysis of Soil and Estimation of Exchangeable Ammonium

The as-received (field-moist) gravimetric water content of all soil samples was measured by drying at 105 °C [34]. During the 2016/17 season, soil samples from inside root exclusion cylinders and from immediately outside were analyzed in the field-moist state for 2M KCl-extractable inorganic N species (no water pre-treatment) with NH_4_-N determined using the modified Berthelot method, and NO_3_-N and NO_2_-N determined by hydrazine reaction and automated color [35,36]. The difference in the concentration of inorganic N species in soil from within the root exclusion cylinders and from immediately outside was used as an indication of the capacity of cotton roots to take up N. Exchangeable NH^4^-N and NO_3_-N adsorbed to mineral exchange sites in soil sampled from within the cylinders was estimated by subtracting results for 2M KCl-extractable N species in water extracts (in solution or loosely bound) [37] from soil 2M KCl results [34].

### 2.4. Irrigation Management and Crop Growth Analysis

Irrigation of the cotton crop at CLP in 2016/17 was scheduled using the soil water deficit, monitored with a neutron probe moisture meter. The crop received a total of 848 mm of water during the growing cycle (450 mm of rain and 398 mm of irrigation). At NAS, the crop received a total of 732 mm of water during the growing cycle (535 mm of rain and 197 mm of irrigation). Irrigation in 2017/18 at SAT (175 mm of rain and 160 mm of irrigation; total: 335 mm) was less frequent and more intense than at INT (175 mm of rain, and 230 mm of irrigation; total: 405 mm), as the site had just been converted from furrow to overhead irrigation and the traveling irrigator was shared with an adjacent maize crop. The overhead irrigation system at INT had been established for 30 years.

Crop development at all sites was monitored by calculating the number of degree-days required for the cotton crop to reach a specific growth phase. Daily air temperature records from weather stations available at the farms were used to calculate the accumulated heat units above 14.7 °C, this being the minimum temperature required for the growth and development of cotton [38], as shown by Equation (1):

(1) $$DD=\frac{\left(T_{MAX}-14.7\right)+\left(T_{MIN}-14.7\right)}{2}$$

where *DD* is degree-days, *T_MAX_* and *T_MIN_* are maximum and minimum air temperatures, and 14.7 is the baseline temperature below which cotton plants do not grow (all in degrees Celsius).

The actual yield of cotton plants within each treatment block was calculated after the cotton crop had been defoliated. The number of plants within each plot was recorded (four plots per treatment), then all plants along a 1 m segment of each row of cotton located within each plot were manually harvested [39]. Lint was manually removed and the average lint yield per treatment was calculated and reported as bales per ha. In Australia, 1 bale of cotton equates to 227 kg of lint.

### 2.5. Data Processing and Statistical Analyses

All soil chemical data were calculated as mg kg^−1^ on a dry mass basis. A one-way analysis of variance with a pair-wise mean post hoc test was used to compare treatments within sampling times, and sampling times within treatments (SigmaPlot Version 12 Systat Software Inc, Palo Alto, CA, USA). Data that could not be normalized using log_10_ transformation were analyzed using the Kruskal–Wallis one-way analysis of variance on ranks, with a pair-wise median post hoc test [40]. A mass balance method was used to compare supplied N and recovered N at each of the five sampling dates over the two cotton growing seasons. At each sampling date, recovered N (total dissolved N (TDN) or dissolved inorganic N extracted from soil) was divided by supplied N (N applied initially as urea plus TDN or dissolved inorganic N extracted from unfertilized soil), and expressed as a percentage.

## 3. Results

### 3.1. Seedling Emergence and Soil Inorganic Nitrogen Supply

**Figure 1:**
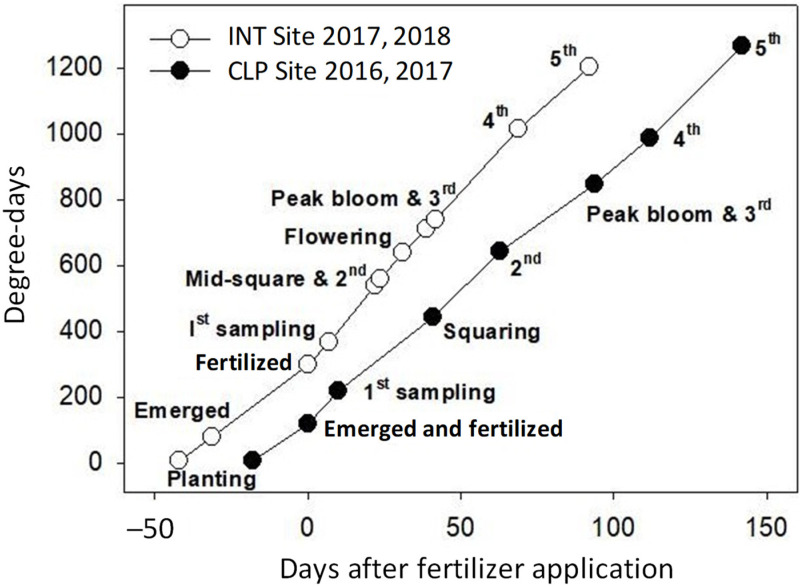
Development of the cotton crop in the 2016/17 (CLP) and 2017/18 (INT) growing seasons. Time taken for the crop to reach key growth stages (degree-days) was plotted against time (days) after fertilizer application. Numerals indicate the soil sampling sequence. Development and sampling at CLP was similar at NAS, and development and sampling at INT was similar at SAT.

Seedling emergence was rapid in 2016/17, with cylinder insertion and fertilizer application occurring 14 (CLP) and 16 (NAS) days after sowing, when seedling density exceeded the minimum required for a commercially viable crop (Table 2). Cold, dry conditions in 2017/18 slowed emergence, delaying cylinder insertion and fertilizer application until 41 days after sowing (Figure 1). Emergence was slowest at the dry-sown SAT site, which had recently been converted from furrow to overhead irrigation. Higher minimum temperatures experienced by the crop after fertilizer application (higher degree-days in Figure 1) enhanced the rate of plant development relative to the 2016/17 season.

**Figure 2:**
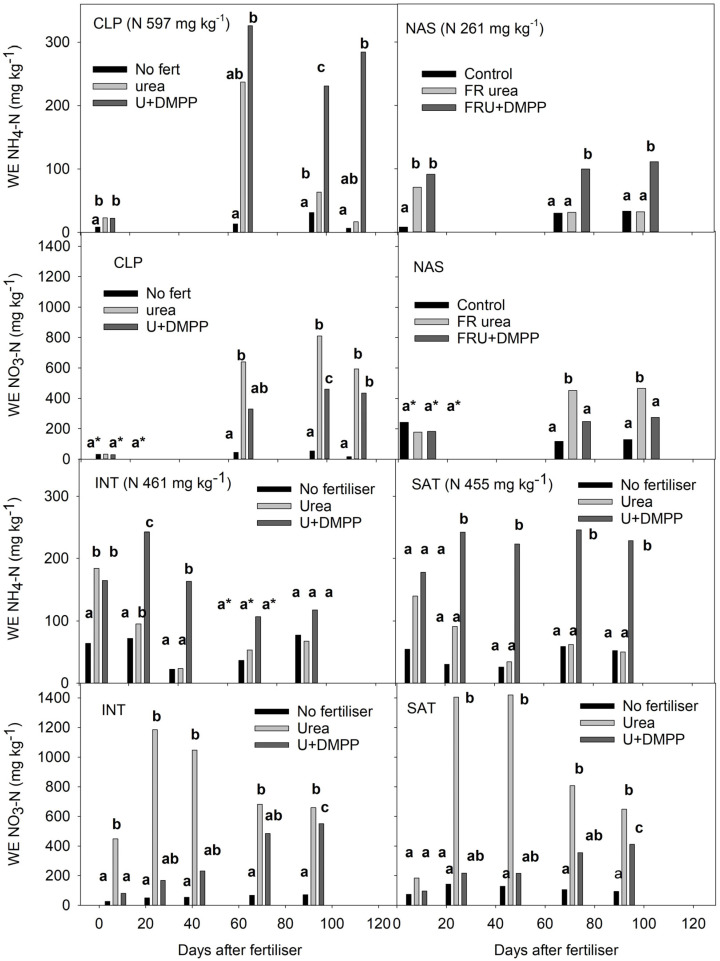
Water extract 2M KCl-extractable NH_4_-N and NO_3_-N (shown as WE NH_4_-N and WE NO_3_-N, respectively) in soil from root exclusion cylinders fertilized with urea, DMPP-coated urea (U+DMPP), or unfertilized (control) at CLP and NAS in 2016–2017, and at INT and SAT in 2017–2018. Rate of urea N banded into soil within root exclusion cylinders is given in brackets. Bars within each treatment with the same letters are not significantly different (*p* > 0.05). Asterisks indicate that results for the pairwise comparison are not reliable (power of performed test < 0.80).

In both seasons, water extract 2M KCl NH_4_-N concentrations remained higher for longer after applying DMPP-coated urea (Figure 2). The inhibition of nitrification reduced NO_3_-N significantly below urea only concentrations across all sites. In the 2016/17 season, the concentration of NH_4_-N recovered 66 days after urea and DMPP-coated urea application (second sampling) remained well below the amount of N applied as urea (597 mg N kg^−1^ soil at CLP and 261 mg kg^−1^ at NAS). After urea application at CLP, NO_3_-N recovered at 66, 97, and 115 days exceeded the amount of N fertilizer applied (Figure 2). Recovered NH_4_-N and NO_3_-N in the unfertilized soil at CLP were consistently low. Data for NO_2_-N have not been included as most recoveries were below the lowest level of reporting. In the 2017/18 season, the frequency of soil sampling was increased within the first 50 days after fertilizer application, and nitrate was analyzed in the presence of nitrite (NO*x*-N). Within 20 days of applying urea and DMPP-coated urea, NH_4_-N recoveries remained well below the applied N rate at both sites (Figure 2), and NO*x*-N recoveries substantially exceeded the applied N rate after urea application only. Within the first 50 days after applying DMPP-coated urea NO*x*-N recoveries remained substantially below the applied N rate, only marginally exceeding it by days 71 and 92. The recovery of NO_3_-N from unfertilized soil was consistently low at both sites.

In the 2016/17 season, at 10 and 66 days after applying urea at CLP (8 days only at NAS), the concentration of 2M KCl-extractable NH_4_-N in soil (no water pre-treatment) was significantly higher than in water extracts (water pre-treatment; Table 3). These observations suggest some of the fertilizer N had bound to soil mineral exchange sites. Coating urea with DMPP delayed the release of exchangeable NH_4_-N (positive values in Table 3) There was no evidence of exchangeable NO_3_-N (water extract NO_3_-N higher than or equivalent to soil NO_3_-N; Table 3), which was as expected as this ion does not bind strongly to soil mineral exchange sites [41].

### 3.2. Recovery of Inorganic Nitrogen Species from Inside and Outside Root Exclusion Cylinders

**Figure 3:**
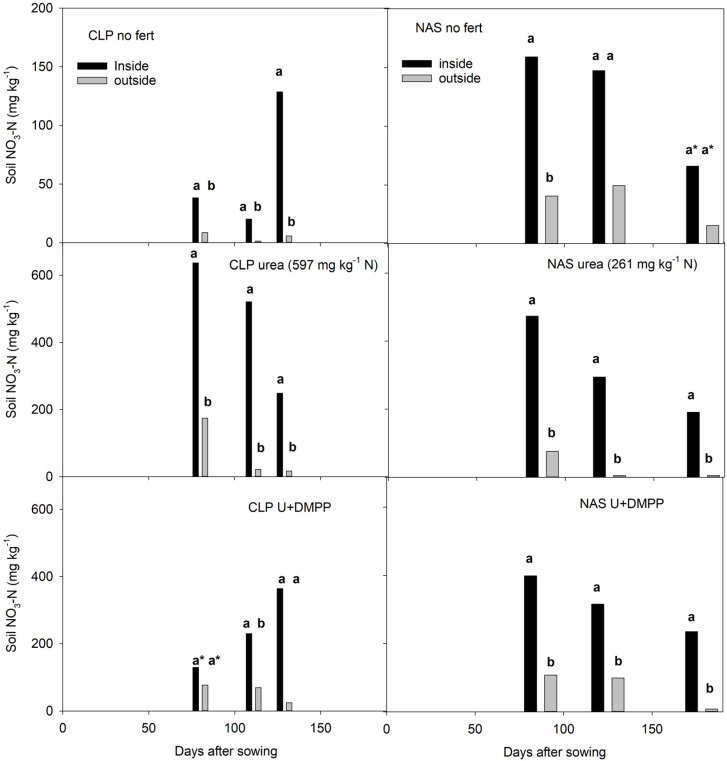
Soil 2M KCl-extractable NO_3_-N inside and outside root exclusion cylinders adjacent to the plant row of unfertilized, urea, and DMPP-coated urea soil at CLP and NAS sampled at key growth stages over the 2016/17 season. Results for soil NH_4_-N are not included as most were below the lowest level of reporting. Numbers in brackets indicate the N application rate per kg of soil. Bars within each sampling time (days after sowing) with the same letters are not significantly different (*p* > 0.05). Asterisks indicates results for the pairwise comparison are not reliable (power of performed test < 0.80).

In the 2016/17 season at CLP, soil 2M KCl-extractable NO_3_-N recovered from inside root exclusion cylinders in unfertilized soil, and after applying urea, remained consistently higher than immediately outside the cylinders (Figure 3), implying roots were actively taking up N. At NAS, inside-cylinder values were consistently higher after urea application, but they were not significantly different in unfertilized soil, which may reflect the difficulty the dry-sown seedlings encountered during emergence. Emerged plants were dry and shriveled and did not fully recover until cut-out (growth phase when floral initiation ceases [42]). Data for soil NH_4_-N are not included as results for both sites were mainly below the lowest limit of reporting. After applying DMPP-coated urea at CLP, the recovery of NO_3_-N from inside cylinders increased over time, but the difference in concentration inside and outside was only significant at 115 days after fertilizer application (Figure 3). At NAS, the recovery of NO_3_-N from soil outside the cylinders after applying urea and DMPP-coated urea was consistently lower than from inside, implying roots were actively taking up N. The inside-cylinder concentration of NO_3_-N within 71 days of applying both urea and DMPP-coated urea was higher than the rate of N applied as urea, with the increase equivalent to the amount of NO_3_-N recovered from the unfertilized soil (Figure 3).

### 3.3. Supplied and Recovered Nitrogen Species

**Figure 4:**
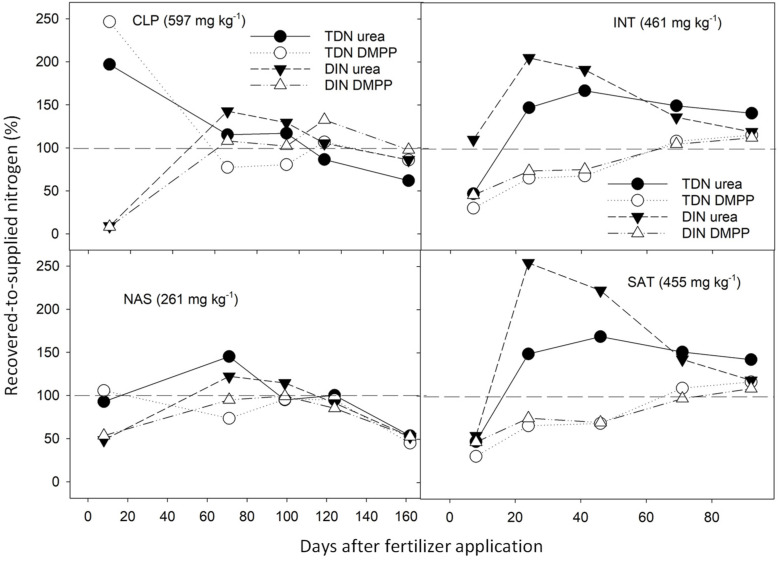
Total dissolved N (TDN) and dissolved inorganic N (DIN) in soil from root exclusion cylinders (recovered N), divided by supplied N (that is, N applied as urea or DMPP-coated urea plus TDN or DIN in unfertilized soil), expressed as a percentage. Dashed line is 100%. Days after fertilizer application refers to the time when cylinders were removed from the soil in 2016–2017 (CLP and NAS), and 2017–2018 (INT and SAT), respectively.

In both seasons, a peak in N within the first 30 days after applying urea and DMPP-coated urea was evident at the CLP, INT, and SAT sites (Figure 4), with recovered TDN over twice the concentration of supplied N. The peak in TDN at CLP in 2016/17 occurred within the first 10 days, whereas in 2017/18 at INT and SAT the peak was delayed until the second sampling 24 days after applying both urea and DMPP-coated urea. The change in analytical method from oxidative combustion, infrared detection to alkaline potassium peroxodisulfate digestion, flow injection automated color may have affected results as high concentrations of dissolved organic matter interfere with the peroxodisulfate digestion [31]. Water extracts prepared from INT and SAT soil within the first 24 days after applying urea and DMPP-coated urea strongly absorbed ultraviolet light at the 254 nm wavelength (a surrogate for heterocyclic dissolved organic matter [43]), and an organic precipitate developed after the addition of 4M KCl [29].

The microbial mineralization of high concentrations of dissolved organic N released after applying urea at INT and SAT was consistent with the increase in dissolved inorganic N evident from 24 days after urea application (Figure 4). Recovered dissolved inorganic N at INT and SAT after 24 and 46 days was well above the supplied N, and well above the TDN. After applying DMPP-coated urea at INT and SAT, recovered dissolved inorganic N remained below supplied N until day 71, and only marginally exceeded supplied N. DMPP may inhibit a wider range of soil microbes than was previously considered (e.g., [44,45]). The compound is known to inhibit nitrifying microbes [46], and our results indicate it also inhibits the heterotrophic microbial mineralization of released organic N.

### 3.4. Crop Performance and Cotton Lint Yield

In the 2016/17 season at CLP, the scheduling of irrigation with the soil water deficit enhanced the growth and development of the cotton crop, with an average of 14 plants per meter row, producing the equivalent of 12.4, 13.7, and 14.1 bales ha^−1^ of lint (no fertilizer, urea, and DMPP-coated urea, respectively). The difference in lint yield was not statistically significant (*p* = 0.301, *n* = 12), despite the higher availability of NO_3_-N after urea and DMPP-coated urea application (Figure 2). At the NAS site, 13 plants per meter row yielded the equivalent of 5.9, 6.1, and 6.9 bales ha^−1^ of lint (no fertilizer, urea, DMPP-coated urea, respectively). Treatment differences in lint yield at NAS were also not statistically significant (*p* = 0.574, *n* = 12), with the stunted early growth of the crop reducing lint production to about half the yield produced at CLP. The cold, dry start to the 2017/18 growing season was also reflected in the lower harvest plant density of 8, 10, and 10 plants m^−1^ at INT (no fertilizer, urea, and DMPP-coated urea, respectively; *p* = 0.198, *n* = 12), and 9, 10, and 10 plants m^−1^ at SAT (no fertilizer, urea, DMPP-coated urea, respectively; *p* = 0.251, *n* = 12). The more regular irrigation regime at INT enabled the crop to recover to produce the equivalent of 11.6, 12.6, and 13.6 bales ha^−1^ of lint (no fertilizer, urea, DMPP-coated urea, respectively; *p* = 0.424, *n* = 12). The crop grown at SAT did not recover to the same extent, producing 7.9, 8.2, and 8.9 bales ha^−1^ of lint (no fertilizer, urea, DMPP-coated urea, respectively; *p* = 0.163, *n* = 12). As in the 2016/17 season, the greater availability of NO_3_-N after applying urea and DMPP-coated urea at INT and SAT did not significantly improve the agronomic response or lint yield at harvest.

## 4. Discussion

### 4.1. Capacity of Cotton Roots to Utilize Fertilizer Nitrogen

Results indicated cotton roots actively take up N from concentrated subsurface banded urea within the first 70 days after applying urea fertilizer. Roots are the major sink for N until floral initiation and may constitute 20% to 25% of crop dry matter until boll formation [20]. Low N fertilizer use efficiencies (e.g., <40%) estimated for cotton in earlier studies (e.g., [1,2,29,47,48]) may be due to the high concentration of soil organic N released within the first 20 days after applying urea and urea-containing fertilizers. Our experiments showed that the release of organic N coincided with an increase in exchangeable soil NH_4_-N. We postulate the sorption of ^15^N to soil mineral exchange sites, and the desorption and microbial mineralization of unlabeled soil organic N after applying a concentrated band of isotopically labeled ammonia-based fertilizer, will dilute the pool of ^15^N available for root uptake [49], reducing N fertilizer use efficiency. Applying repeated, low-rates of ammonia-based fertilizer scheduled with crop demand for N [19], or allocating more fertilizer bands per plant row for single high-rate applications may improve N fertilizer use efficiency by reducing the release of soil organic N.

### 4.2. Mechanism of Soil Organic Nitrogen Release

Studies with isotopically labeled N fertilizer indicate the release of dissolved organic matter (DOM) is greater after the application of ^15^N ammonia-based fertilizer than after ^15^N nitrate [50]. Some researchers postulate the rapid rise in soil pH driven by urease enzymes converting urea to ammonium induces the chemical hydrolysis of organic compounds (alkaline hydrolysis) [51,52]. The subsequent reduction in soil pH due to nitrification theoretically re-polymerizes, or precipitates, organic compounds, consequently reducing the concentration of DOM. However, no reduction in DOM was detected within 20 days of anhydrous ammonia application, after the soil pH was chemically lowered [53]. Our alternative hypothesis is that high concentrations of ammonia-based fertilizer drive the sorption of NH_4_^+^ onto soil mineral exchange sites [54], releasing previously sequestered DOM. Fertilizer-driven NH_4_^+^ sorption should be greater in summer cropping systems, as the rate of urea hydrolysis increases significantly with a rise in temperature (from 15° to 35 °C; Lei et al. [55]). In our experiments, the release of high concentrations of soil organic N occurred after applying both urea and DMPP-coated urea. Coating urea granules with DMPP inhibited both nitrification and the mineralization of organic N, reducing the supply of NO_3_-N available for root uptake. Ammonium-N concentrations remained higher for longer, driving the sorption of fertilizer-derived NH_4_^+^ onto soil mineral exchange sites (higher exchangeable NH_4_-N in Table 3) with the previously sequestered desorbed dissolved organic N prone to leaching [56]. This may explain why DMPP reduces nitrous oxide emissions and nitrate loss in broadacre agricultural systems, without necessarily improving crop yield and N fertilizer use efficiency [2,15,16,57].

### 4.3. Implications for Soil and Nitrogen Management in Cotton

In the eastern region of subtropical Australia (latitude range: 20° to 30° S, longitude range: 140° to 150° E), cotton is grown on predominantly clay soils in rotation (e.g., winter or summer cereals) or continuously. Granulated urea and anhydrous ammonia are the preferred N fertilizer sources [58]. Total soil organic carbon in the Black Vertosols of SE Queensland (Australia) has declined to between 24% and 33% of original levels [59,60]. Dissolved organic N may account for 40% of total N lost from subtropical Australian soils cultivated to cotton [61], and for more than 50% from tropical soils cultivated to sugarcane [62]. The high concentration of urea applied to these crops before or at planting may drive the sorption of NH_4_^+^ onto soil exchange sites, releasing previously sequestered DOM. Reducing the concentration of urea by increasing the number of fertilizer bands per plant row, or synchronizing fertilizer N application with high crop N demand [18,63] may reduce NH_4_^+^ sorption and the release and loss of DOM [64], improving crop N fertilizer use efficiency and soil carbon sequestration.

## 5. Conclusions

Shallow subsurface application of ammonia-based fertilizer placed in concentrated bands at rates equal to or above 460 mg N kg^−1^ of soil at the start of the cotton growing season released high concentrations of previously sequestered dissolved organic N from soil. The microbial mineralization of released organic compounds increased plant-available N to 100% above N supplied as urea and from the unfertilized soil. Coating urea with the nitrification inhibitor DMPP (3,4-dimethylpyrazole phosphate) increased the concentration of NH_4_-N bound to soil exchange sites and inhibited the microbial decomposition of dissolved organic N. Our results suggest the release of previously sequestered organic N within 30 days of applying a concentrated band of urea distorts the N fertilizer use efficiency calculation. Fertilizer application methods that reduce the concentration of banded urea in soil, and which improve the timing of N supply with crop N demand, may improve crop N uptake, N fertilizer use efficiency, and carbon sequestration.

## Figures and Tables

**Table 1:** Characterization of soil in four cotton fields, sampled from two depths (0–150 mm and 150–300 mm). CLP and NAS were sampled in October 2016, INT and SAT were sampled in November 2017. TDN is total dissolved N and SBD is soil bulk density. Exchangeable (Exch) cations were extracted after pre-treatment for soluble salts.

Site/Depth	pH_1:5_	EC	TDN	Exch Ca	Exch Mg	Exch Na	Exch K	CEC	SBD
Unit	-	dS m^−1^	mg kg^−1^	mEq 100 g^−1^	mEq 100 g^−1^	mEq 100 g^−1^	mEq 100 g^−1^	mEq 100 g^−1^	g cm^−3^
CLP, 0–150	8.37	0.09	46.4	13.20	12.60	0.64	0.51	26.95	1.00
CLP, 150–300	8.95	0.10	44.4	12.33	13.89	1.33	0.22	27.78	1.01
NAS, 0–150	9.36	0.31	130.5	12.93	12.45	2.43	0.77	28.58	1.04
NAS, 150–300	9.55	0.42	116.9	11.01	13.94	4.27	0.46	29.58	1.02
INT, 0–150	8.40	0.22	33.2	17.48	16.38	0.17	0.68	34.72	0.98
INT, 150–300	8.33	0.24	44.4	16.60	15.47	0.19	0.39	32.65	1.05
SAT, 0–150	7.98	0.43	55.7	18.82	15.60	0.17	0.84	35.42	1.00
SAT, 150–300	8.25	0.41	46.3	19.31	15.28	0.22	0.56	35.37	1.06

**Table 2:** Crop sowing date, date of nitrogen (N) fertilizer application, and N fertilizer rate per root exclusion cylinder applied at NAS and CLP in 2016, and at INT and SAT in 2017.

Season	-	2016–2017	2016–2017	2017–2018	2017–2018
Experimental Site	Units	CLP	NAS	INT	SAT
Planting	-	21 October 2016	14 October 2016	1 November 2017	2 November 2017
Fertilizer application	-	4 November 2016	30 October 2016	12 December 2017	12 December 2017
N rate/root exclusion cylinder	mg N kg^−1^ soil	597	261	461	455
Field-equivalent N fertilizer rate	kg N ha^−1^	142	129	147	147

**Table 3:** Exchangeable NH_4_-N and NO_3_-N in soil from root exclusion cylinders after applying urea and DMPP-coated urea in the 2016/17 season. Results for 2M KCl extraction of water extracts (soluble or loosely bound N species) were subtracted from 2M KCl soil extraction results. Column values with the same letter are not significantly different at a 5% probability level.

Sampling Time	CLP, NH_4_-N (mg kg^−1^)			CLP, NO_3_-N (mg kg^−1^)		
(Days)	No-Fertilizer	Urea	Urea + DMPP	No Fertilizer	Urea	Urea + DMPP
10	−3.5 a	448.0 a	521.1 a	−11.4 a	−11.5 a	−8.6 a
66	−13.5 b	271.8 ab	391.0 a	−5.9 ab	−2.3 a	−46.5 a
97	−31.7 c	−18.0. b	110.8 a	−3.1 ab	−247 8 a	−228.4 a
115	−6.4 a	−17.1 b	75.0 a	5.4 b	81.9 a	−73.3 a
152	−0.4 a	0.0 b	−2.1 a	4.3 b	−142.1 a	−141.4 a
**Sampling Time**	**NAS, NH_4_-N (mg kg^−1^)**			**NAS, NO_3_-N (mg kg^−1^)**		
**(Days)**	**No-Fertilizer**	**Urea**	**Urea + DMPP**	**No Fertilizer**	**Urea**	**Urea + DMPP**
8	−7.4 ab	346.0 a	436.2 a	−79.3 a	−29.6 a	−11.0 a
71	44.8 ab	−31.3 ab	48.9 ab	73.5 a	−89.7 a	−41.3 a
99	−32.4 a	−32.1 b	112.8 ab	41.9 a	110.0 a	−73.4 a
124	−14.4 ab	−13.8 ab	10.8 b	33.3 a	−42.2 a	22.3 a
162	−1.6 b	−1.9 ab	0.0 b	9.1 a	27.1 a	34.1 a

## Data Availability

Electronic data associated with this study can be requested from Dr Diogenes L. Antille (dio.antille@csiro.au) and Dr Pamela A. Pittaway (pam.pittaway@usq.edu.au).
